# Bioactivity-guided fractionation of *Helicteres angustifolia* L. extract and its molecular evidence for tumor suppression

**DOI:** 10.3389/fcell.2023.1157172

**Published:** 2023-06-22

**Authors:** Kejuan Li, Shuang Sun, Long Xiao, Zhenya Zhang

**Affiliations:** ^1^ College of Life Science, Sichuan Normal University, Chengdu, China; ^2^ Graduate School of Life and Environmental Sciences, University of Tsukuba, Tsukuba, Japan

**Keywords:** *H. angustifolia*, lung cancer, reactive oxygen species, Nrf2, metastasis

## Abstract

*Helicteres angustifolia* L. (*Helicteres angustifolia*) has been commonly used in folk medicine to treat cancer; however, its mechanisms of action remain obscure. In our earlier work, we reported that aqueous extract of *H. angustifolia* root (AQHAR) possesses attractive anticancer properties. In the present study, we isolated five ethanol fractions from AQHAR and investigated their therapeutic efficacy in human non-small cell lung cancer (NSCLC) cells. The results showed that among the five fractions, the 40% ethanol fraction (EF40) containing multiple bioactive compounds exhibited the best selective killing effect on NSCLC cells with no obvious toxicity to normal human fibroblasts. Mechanistically, EF40 reduced the expression of nuclear factor-E2-related factor 2 (Nrf2), which is constitutively expressed at high levels in many types of cancers. As a result, Nrf2-dependent cellular defense responses are suppressed, leading to the intracellular accumulation of reactive oxygen species (ROS). Extensive biochemical analyses revealed that EF40 caused cell cycle arrest and apoptosis through activation of the ROS-mediated DNA damage response. Furthermore, treatment with EF40 compromised NSCLC cell migration, as evidenced by the downregulation of matrix metalloproteinases (MMPs) and heterogeneous nuclear ribonucleoprotein K (hnRNP-K). *In vivo* studies using A549 xenografts in nude mice also revealed significant suppression of tumor growth and lung metastasis in the treated group. We propose that EF40 may serve as a potential natural anti-NSCLC drug that warrants further mechanistic and clinical attention.

## Introduction

Lung cancer is the major cause of cancer-related deaths worldwide in humans (Chen et al., 2016; Siegel et al., 2020). Among all cases, non-small cell lung cancer (NSCLC) comprises almost 85%, with only approximately a quarter of patients diagnosed with disease that is curative (Zappa and Mousa, 2016). To date, platinum drugs (mainly cisplatin and its derivatives) are still used as first-line chemotherapeutic treatments. However, these drugs have a large number of side effects, including nephrotoxicity, cytotoxicity, and neurotoxicity (Yuan et al., 2019). Thus, novel drugs are urgently needed to improve cancer treatment outcomes.

Since long ago, herbal medicines have been used as cancer therapeutics with proven efficacy and safety. In fact, over 60% of the currently used anticancer agents originate from natural sources in one way or another (Cragg and Newman, 2009). *Helicteres angustifolia* (*Helicteres angustifolia*), a medicinal herb belonging to the Sterculiaceae family, is commonly used in folk medicine to treat various diseases, including cancer. Modern pharmacological research has demonstrated its anti-hepatic fibrosis, antiviral, antidiabetic, and immunomodulatory activities (Huang et al., 2012; Huang et al., 2013; Hu et al., 2016; Sun et al., 2018). Several bioactive compounds, including naphthoquinone, sesquiterpenoid quinones, flavonoids, lignans, triterpenoids, steroids, and alkaloids, have been identified in this plant (Wang and Liu, 1987; Chen et al., 1990; Chen et al., 1994; Chin et al., 2006; Pan et al., 2008; Wang et al., 2012). In addition, chloroform, ethanol, and methanol extracts of *H. angustifolia* root exhibit cytotoxic activity against various cancer cells (Lu et al., 2000; Chen et al., 2006; Pan et al., 2008; Wang et al., 2012).

Reactive oxygen species (ROS), mainly comprising hydroxyl radicals, superoxide anions, hydrogen peroxide, and singlet oxygen, potentially negatively affect organisms. Oxidative stress resulted from an imbalance between the production and elimination of cellular ROS, results in irreversible oxidative damage and, thus, to cell death (Lawless et al., 2010). Many anticancer drugs have been developed to induce oxidative stress as part of their mode of action for killing cancerous cells. However, cancer cells may become resistant to drugs by augmenting their antioxidant capacity through the deregulation of nuclear factor-E2-related factor 2 (Nrf2). Nrf2 is an important transcription factor that regulates redox homeostasis in cells. Nrf2 recognizes antioxidant response elements (AREs) located in the promoters of a battery of genes encoding antioxidant and detoxification enzymes (Maher and Yamamoto, 2010; Pi et al., 2010). Under oxidative stress, Nrf2 activates downstream cellular protective genes to protect the cells from ROS-induced damage. Compared to normal cells, cancer cells produce ROS more abundantly and adapt to elevated oxidative stress due to the upregulated expression of the Nrf2 protein. Aberrant activation of Nrf2-ARE signaling correlates with tumor initiation, progression, and resistance to therapy (Lau et al., 2008; Hayes and McMahon, 2009; Kensler and Wakabayashi, 2010). Therefore, Nrf2 inhibition has the potential to eliminate tumors *via* oxidative stress modulation while sparing normal tissues.

We have recently found aqueous extract of *H. angustifolia* root (AQHAR) inhibits tumor growth by inducing ROS stress and DNA damage (Li et al., 2015; Li et al., 2016a). We separated AQHAR into ethanol fraction (EF) and water fraction (WF) using 80% ethanol and found that EF, as the main contributor to AQHAR toxicity, exhibited a significantly higher anticancer activity than WF (Li et al., 2016b). In this study, to explore the key therapeutic components in *H. angustifolia*, we further purified EF into five fractions and examined their cytotoxicity in NSCLC cells and normal lung fibroblasts. The most effective fraction, the 40% EF (EF40), was selected for further study of its underlying molecular mechanisms. We demonstrated that EF40 selectively reduced the protein levels of Nrf2 in NSCLC cells and triggered ROS-mediated cell death. Furthermore, the antimetastatic potential of EF40 was investigated using both *in vitro* and *in vivo* assays.

## Materials and methods

### Preparation of the AQHAR and its EtOH fractions

*Helicteres angustifolia* roots were collected from a rural area of Vientianei, Laos (18°447′N, 102°4944′E) in August 2013. Botanical identification was performed at the Kunming Institute of Botany, Chinese Academy of Sciences, Kunming, China. The dry roots of *H. angustifolia* (200 g) were ground into a fine powder (diameter <0.6 mm) and extracted twice with 2 L distilled water for 24 h. The filtrates of the two extractions were combined and condensed at 50°C in a rotary vacuum evaporator, after which the concentrated extract was freeze-dried to obtain an 8.4% AQHAR extract and stored at −20°C until further use.

EF was extracted from AQHAR using 80% ethanol as previously described (Li et al., 2016b). EF was then dissolved in deionized water, loaded onto HP20 macroporous resin (Mitsubishi Chemical Co., Ltd., Tokyo, Japan), and sequentially fractioned with pure water and ethanol (20%, 40%, 60%, and 80%), and each eluent obtained successively was freeze-dried to powder and labeled EF0, EF20, EF40, EF60, and EF80, respectively (as shown in Supplementary Figure S1).

### Liquid chromatography-Orbitrap mass spectrometry (UPLC-Orbitrap-MS) analysis of EF40

EF40 was diluted with 50% methanol to obtain a solution at a concentration of 2 mg/mL, and the samples were filtered through a 0.22 μm filter prior to UPLC-Orbitrap-MS detection. UPLC analysis was performed on an Vanquish UHPLC (Thermo Fisher Scientific, San Jose, CA, United States). Reversed-phase separation was performed using an ACQUITY UPLC High Strength Silica T3 column (2.1 mm × 100 mm, 1.8 μm; Waters, Milford, MA, United States) with a constant flow rate of 0.3 mL/min at 25°C. Water (containing 0.1% formic acid, mobile phase A) and methanol (mobile phase B) were used as the mobile phases and eluted with a linear gradient elution of 5%–100% A at 0–25 min. The injection volume was 0.5 μL.

To detect the chemical composition of EF40, an Orbitrap Exploris 120 mass spectrometer (Thermo Fisher Scientific, San Jose, CA, United States) equipped with a heated-electrospray ionization source in both positive and negative ionization modes was used with the following parameters: ion transfer tube temperature, 320°C; vaporizer temperature, 350°C; sheath gas flow rate, 50 arb; auxiliary gas flow rate, 15 arb; radio frequency level, 70%; and spray voltage, 3.5 kV/− 2.5 kV. The peaks were then matched with the database of traditional Chinese medicine (mzCloud, mzVault, and ChemSpider) to obtain qualitative results using the Xcalibur 4.4 software (Thermo Fisher Scientific).

### Cell culture, transfection, and treatment

The human NSCLC cell lines A549 and NCl-H1299 (H1299), fetal lung fibroblast MRC-5, and normal fibroblast TIG-3 were obtained from the Cell Resource Center for Biomedical Research, Aging and Cancer (Tohoku University, Japan) and cultured in Dulbecco’s modified Eagle’s medium (DMEM) supplemented with 10% fetal bovine serum, 100 U/mL penicillin, and 100 μg/mL streptomycin. All the cells were incubated in a stable environment with 5% CO_2_/95% air at 37°C in a humidified incubator. Cells were transfected with HyPer-nuc plasmid (Evrogen, Moscow, Russia) with Fugene HD (Roche Applied Sciences, Basel, Switzerland) according to the manufacturer’s instructions. Transfected cells were selected and maintained in a medium supplemented with G418 (Thermo Fisher Scientific). Unless otherwise mentioned, cells were treated with EF40 or sodium arsenite (ASN; Wako, Osaka, Japan) at approximately 60% confluency. Morphological observations were recorded after treatment using a phase-contrast inverted microscope fitted with a digital camera (Digital Sight DS-L1; Nikon, Tokyo, Japan).

### Cell viability assay

The cell viability assay was performed using the 3-(4, 5- dimethylthiazol-2-yl)-2, 5-diphenyltetrazolium bromide (MTT) assay as previously described (Li et al., 2016a) or with the Cell Counting Kit-8 according to the manufacturer’s protocol (Dojindo, Kumamoto, Japan). Briefly, the cells (3 × 10^3^ cells well^−1^) were seeded in 96-well plates and allowed to adhere overnight. The cells were then exposed to the obtained EFs for 24 h. The absorbance at 450/690 nm was measured using a microplate reader (Infinite M200 PRO; Tecan, Männedorf, Switzerland). All samples were assayed in triplicates.

### Colony formation assay

The colony formation assay was performed as previously described (Yu et al., 2020). Briefly, A549 and H1299 cells were seeded in 6-well plates at a density of 500 cells/well and allowed to adhere for approximately 12 h. The cells were then treated with DMEM containing 25 μg/mL EF40 for 6 h, followed by culturing in normal medium for 10 days. Cells treated with normal medium were used as the controls. After treatment, cells were fixed with a methanol/acetone (v/v, 1:1) mixture for 10 min and stained overnight with 0.5% crystal violet solution (Wako). The number of colonies was counted, and the mean number of colonies was calculated from three independent experiments.

### 5-Ethynyl-2′-deoxyuridine (EdU) proliferation assay

Cell proliferation was detected using a Click-iT™ Plus EdU Cell Proliferation Kit (Thermo Fisher Scientific, Waltham, MA, United States) according to the manufacturer’s instructions. Briefly, A549 and H1299 cells (1 × 10^5^ cells well^−1^) were seeded onto glass coverslips in 12-well culture dishes. After EF40 treatment (25 μg/mL, 24 h), the cells were incubated with 10 µM EdU for 1 h before fixation with 4% paraformaldehyde at room temperature (RT) for 15 min. The fixed cells were then washed with phosphate-buffered saline (PBS) and stained with a 1 ×  Click-iT™ Plus reaction cocktail for 30 min at RT in the dark, followed by counterstaining with Hoechst 33342. After serial washing with PBS, the coverslips were mounted and visualized under a microscope (Axiovert 200 M; Carl Zeiss, Tokyo, Japan). The number of EdU-positive cells was counted in five randomly selected areas and normalized to the nucleus.

### Measurement of reactive oxygen species

Cellular ROS were measured using 2′,7′-dichlorodi hydrofluorescein diacetate (H_2_DCFDA; Molecular Probes, Thermo Fisher Scientific) according to the manufacturer’s protocol. Briefly, the cells (5 × 10^3^ cells well^−1^) were seeded in black 96-well flat-bottom plates with a clear bottom (Thermo Fisher Scientific). After cell adherence, the cells were treated with EF40 (25 and 50 μg/mL) for either 4 h or 24 h, followed by incubation with 10 µM H_2_DCFDA for 30 min. Fluorescence derived from ROS was detected at 485 nm excitation and 535 nm emission wavelengths using a fluorescence microplate reader (InfiniteF200 PRO; Tecan).

To examine mitochondrial ROS levels, cells cultured in poly L-lysine-coated 35-mm glass-bottom dishes (Matsunami, Tokyo, Japan) were exposed to EF40 (25 μg/mL, 24 h), followed by loading with MitoSOX Superoxide Indicator (5 μM; molecular probes) for 20 min at 37°C. After washing twice with PBS, cells were stained with Hoechst 33342 (Thermo Fisher Scientific) for 10 min at 37°C and imaged immediately in phenol red-free medium (Thermo Fisher Scientific) with a DeltaVision deconvolution microscope equipped with a CO_2_ chamber at 37°C (IX71, Olympus, Tokyo, Japan).

### Dual luciferase reporter assay

The OKD reporter plasmid (pOKD-luc; TransGenic, Tokyo, Japan) was used to evaluate Nrf2-ARE signaling activity, according to the manufacturer’s instructions. Briefly, cells (3 × 10^5^ cells well^−1^) were seeded in a 35 mm dish and transiently transfected in triplicate with 2 μg of luciferase construct (pOKD-luc) and 100 ng of control vector oligonucleotide (pRL-TK; Promega, WI, United States) using Fugene HD (Roche). At 24 h post-transfection, cells were exposed to EF40 treatments (25 and 50 μg/mL) for approximately 16 h. Firefly and Renilla luciferase activities were determined using a dual-luciferase reporter assay system (Promega).

### Western blot analysis

Whole-cell lysates were prepared using a radioimmuno precipitation assay lysis buffer (Thermo Fisher Scientific) with freshly added protease inhibitor cocktail (Roche) for 30 min on ice and then centrifuged at 16,000 × g at 4°C for 10 min. The supernatant was collected, and the protein concentration was calculated using a BCA protein assay kit (Thermo Fisher Scientific). Nuclear and cytoplasmic fractions were prepared using NE-PER^®^ Nuclear and Cytoplasmic Extraction reagents (Thermo Fisher Scientific), according to the manufacturer’s instructions. The lysate (20 μg) was heated to 95°C for 5 min, and SDS-PAGE was used to resolve the proteins in the lysate. After electrophoresis, the proteins were electro-transferred to polyvinylidene difluoride membranes (Bio-Rad, Tokyo, Japan) and then immunodetected with antibodies on a Gel Doc XR + system (Bio-Rad) using standard procedures. Detailed information about antibodies and their dilutions are listed in Supplementary Table S3.

### Quantitative polymerase chain reaction (qPCR) analysis

Total RNA was isolated using the RNeasy Plus Mini Kit (Qiagen, Stanford Valencia, CA, United States) and reverse-transcribed according to the protocol of the QuantiTect Reverse Transcription Kit (Qiagen). Real-time qPCR was performed using the SYBR Select Master Mix (Applied Biosystems, Thermo Fisher Scientific) in triplicate on an Eco™ real-time system (Illumina, San Diego, CA, United States). The fold-change in mRNA expression was calculated using the ΔCt method, with the 18S transcript as an internal control. The primer sets used are listed in Supplementary Table S4.

### Fluorescence microscopy imaging

For immunofluorescence, cells (1 × 10^5^ cells well^−1^) were plated on glass coverslips in a 12-well culture dish. After treatment, the cells were washed with cold PBS, fixed with 4% formaldehyde in PBS for 10 min, permeabilized with 0.5% Triton X-100 in PBS for 10 min, and blocked in 2% bovine serum albumin in PBS for 1 h. They were then incubated with primary antibodies (detailed in Supplementary Table S3) overnight at 4°C, followed by incubation with secondary antibodies labelled with Alexa Fluor 488 or 594 at RT for 1 h. Nuclei were counterstained with Hoechst 33342. Coverslips with stained cells were mounted and stored at 4°C in the dark until imaging was performed. Images were quantified using the ImageJ software (National Institute of Health, Bethesda, MD).

Mitochondrial membrane potential was monitored using a JC-1 MitoMP Detection Kit (Dojindo). Cells (2 × 10^5^) were seeded in 35-mm glass bottom dishes and allowed to adhere overnight. After 24 h of EF40 treatment, JC-1 solution was added to the culture medium to a final concentration of 4 μM and then incubated at 37°C for 30 min. After washing twice with culture medium, the cells were live-imaged using the DeltaVision microscope setup outlined above.

### Neutral comet assay

DNA double-strand breaks were quantified using a comet assay system (Trevigen, Gaithersburg, MD, United States) following the manufacturer’s recommendations and as described previously, with slight modifications (Yu et al., 2017). Briefly, cells (2 × 10^5^ cells well^−1^) were seeded in 6-well plates, cultured in the presence or absence of EF40 (25 μg/mL) for 24 h, and then harvested and mixed with 0.5% low melting point agarose (Trevigen). The mixture (50 µL) was spread onto fully frosted glass microscope slides and allowed to solidify at 4°C for 30 min. The slides were then submerged in lysis solution overnight at 4°C and incubated for 30 min in neutral electrophoresis buffer solution. After electrophoresis at neutral pH for 30 min, the slides were washed thrice with neutralizing buffer, dried at RT overnight, and stained with SYBR Green (Thermo Fisher Scientific) for fluorescence microscopy. The resulting images were analyzed using the ImageJ software. DNA damage was assessed by measuring the percentage of DNA in the tail using OpenComet software (v1.3.1).

### Flow cytometric analyses

The cells (1 × 10^5^ cells well^−1^) were seeded into 6-well plates. After 24 h of attachment, the cells were treated with different concentrations of EF40 (0, 10, 25, and 50 μg/mL) for 24 h, followed by harvesting with trypsin-EDTA. For cell cycle analysis, cell pellets were washed with cold PBS, fixed with 70% ethanol on a slow vortex, and kept at −20°C for 48 h. Cells were then stained with Guava Cell Cycle Reagent (Luminex, Austin, TX, United States) and incubated with RNase-A (1 mg/mL; Sigma-Aldrich, Saint Louis, MO, United States) at 37°C for 30 min before analysis using a Guava PCA flow cytometer (Millipore, Burlington, MA, United States). For the measurement of apoptotic cells, cells were harvested with the treatment media and briefly centrifuged to collect both cells and apoptotic bodies. After resuspension in culture medium, the collected cells were incubated with Guava Nexin reagent (Luminex) in the dark for 20 min. Finally, the mixture was analyzed using a Guava flow cytometer. FlowJo software (LLC, Ashland, OR, United States) was used to analyze the flow cytometry data.

### Wound scratch assay

Cells (1 × 10^5^ cells well^−1^) were plated in 6-well plates and allowed to form monolayers by overnight incubation at 37°C. The cell monolayer was scraped with 200 μL pipette tips when the confluency reached 90%. The scratched cells were then rinsed twice with PBS to remove cell debris, and fresh medium supplemented with EF40 (10 and 25 μg/mL) was added. The movement of cells into the scratched area was observed and recorded at 0, 48, and 72 h using a phase-contrast inverted microscope (Nikon, Tokyo, Japan) fitted with a digital camera (Digital Sight DS-L1; Nikon).

### Cell invasion assays

The Transwell invasion assay was used to test the capacity of cells to directionally respond to EF40 treatment. A transwell chamber insert (BD Corporation, Franklin Lakes, NJ, United States) with an 8-μm pore size was used in the present study. Cells (2 × 10^4^) suspended in 100 μL of various conditional serum-free culture media were plated on the top of the invasion inserts. After 6 h of incubation with EF40 at 25 and 50 μg/mL, the cells were wiped from the upper surface with a cotton swab, and the cells that migrated through the inserts were fixed with methanol and stained with 1% crystal violet. The excess stain was removed by rinsing with distilled water. The inserts were air-dried, visualized, and photographed under a microscope (TS100-F; Nikon). At least five random fields for each chamber were counted at ×100 magnification, and the number of invaded cancer cells per chamber was calculated as invasion activity. Each experiment was performed in triplicates.

### Animal experiments

All protocols involving mice were evaluated and approved by the Animal Experiments Committee of the University of Tsukuba (Approval No. 14–375) under veterinary supervision. Female BALB/c nude mice (5-week-old) were obtained from CLEA Japan, Inc. (Tokyo, Japan) and maintained in specific pathogen-free facilities under routine care. To generate tumor xenograft models, nude mice were injected with A549 cells (1 × 10^6^ cells/mouse) in the subdermal space on the medial side of the thigh. Once tumors reached the desired size (∼35 mm^3^), mice were randomly allocated into three groups (*n* = 4) and intraperitoneally treated with PBS, EF40 (100 mg/kg), or EF40 (200 mg/kg) every other day. Tumor formation and body weight were monitored every alternate day. Tumor volume was calculated as V = L × W^2^/2, where L and W are the length and width of the tumor, respectively. At the end of the experiment, the mice were sacrificed, and the tumors were dissected and weighed. Formalin-fixed paraffin-embedded tumor tissue sections were used for further analysis.

For the lung metastasis assay, mice were intravenously injected with A549 cells (1 × 10^5^ cells/mouse) in the tail vein. After inoculation, tumor-injected mice were randomly divided into two groups (*n* = 3): (1) PBS control group and (2) EF40 treatment (100 mg/kg) group. EF40 was orally administered daily for 18 days. On day 18, the mice were sacrificed, and their lungs were excised, removed, and evaluated for tumor metastases on the lung surface.

### Immunohistochemistry (IHC) and terminal deoxynucleotidyl transferase dUTP nick end labeling (TUNEL) assay

The IHC analysis was performed using standard protocols. Briefly, primary tumors were fixed in 10% formaldehyde in PBS at RT followed by routine dehydration, paraffin embedding, and tissue sectioning. After incubation with the primary antibodies (listed in Supplementary Table S3) and horseradish peroxidase-labeled polymer, 4 µm sections were stained with hematoxylin. Apoptosis in paraffin-embedded tissue sections was determined by the TUNEL method using a commercial kit (DeadEnd™ Colorimetric TUNEL System; Promega, Madison, WI, United States), according to the manufacturer’s protocol. All the sections were examined using a NanoZoomer S60 digital slide scanner (Hamamatsu, Shizuoka, Japan). Quantitation was performed using an ImageJ plugin, Trainable Waka Segmentation. The results of quantitative data were converted into an H-Score based on the formula: H-Score = (%low positive × 1) + (%positive × 2) + (%high positive × 3).

### Statistical analysis

All experiments were performed in triplicates, and the data are presented as the mean ± standard deviation (SD). Analysis of variance (ANOVA) was used for multiple comparisons, and the unpaired Student’s t-test was used for two-group comparisons. Significant differences were assumed at ^*^ *p* ≤ 0.05, ^**^ *p* ≤ 0.01, and ^***^ *p* ≤ 0.001. All statistical analyses were performed using GraphPad Prism (version 8.0; San Diego, CA, United States) or SPSS (version 19.0; Chicago, IL, United States).

## Results

### EF40 is cytotoxic to NSCLC cells but not to normal lung fibroblasts

To explore the key anticancer components of EF, we partitioned EF using an HP20 macroporous resin. EF was sequentially eluted with 0% (pure water), 20%, 40%, 60%, and 80% ethanol (Supplementary Figure S1), with yields of 66.7%, 7.2%, 0.6%, 1.7%, and 0.15%, respectively. The cytotoxicity of the above fractions against NSCLC cells (A549 and H1299) and normal cells (TIG-3 and MRC-5) was determined using an MTT assay. As shown in Supplementary Figures S2A, B, EF0 and EF20 showed minor cytotoxic effects on cancerous A549 and H1299 cells, whereas EF40, EF60, and EF80 exhibited significant cytotoxicity in both NSCLC cell lines in a dose-dependent manner. Notably, TIG-3 and MRC5 normal fibroblasts treated with EF40 showed negligible reduction in cell viability (Supplementary Figures S2C, D), indicating the selective killing activity of EF40 specific to cancer cells, but not normal cells.

**FIGURE 1:**
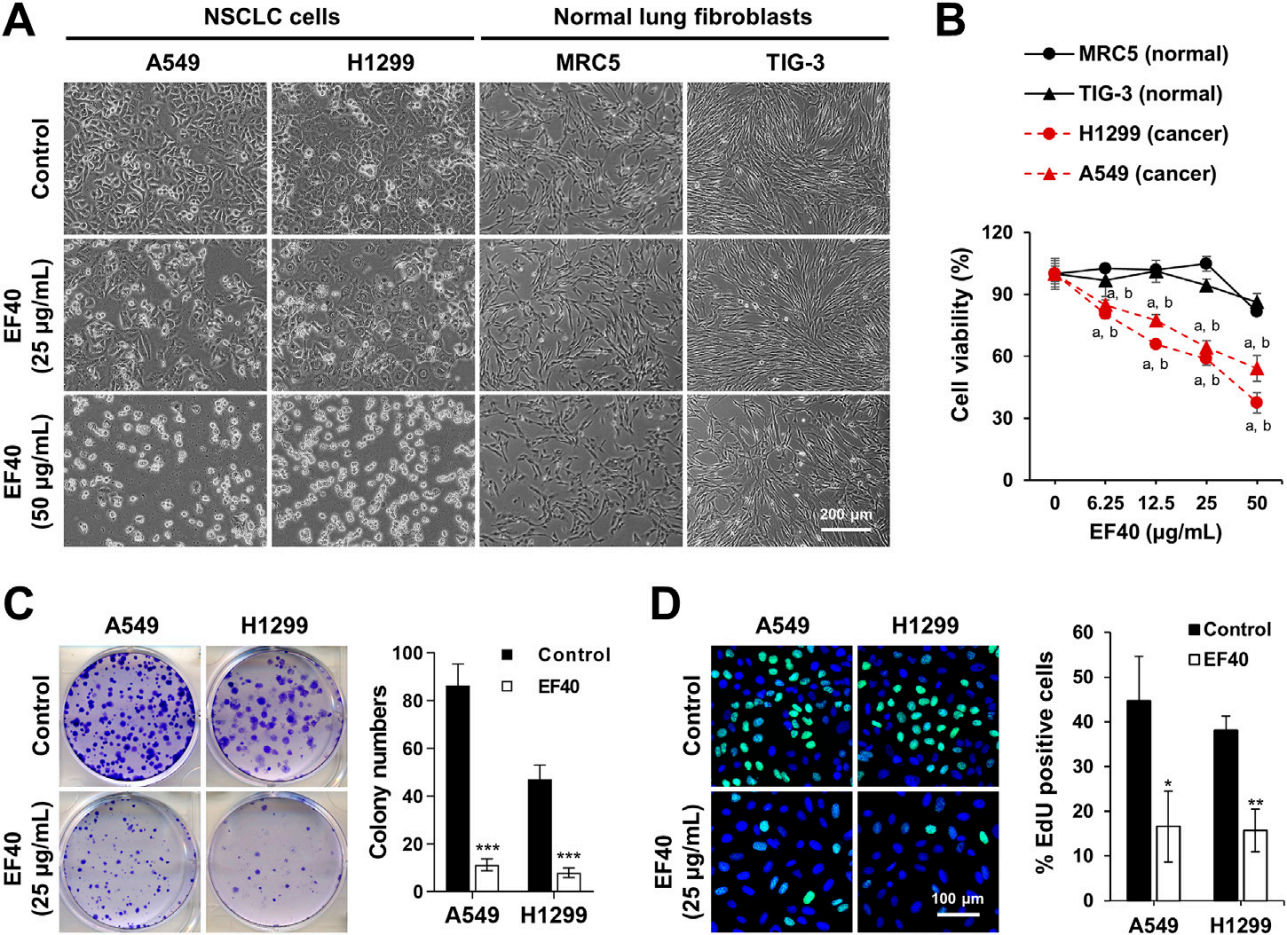
Selective cancer killing activity of EF40 **(A)** Phase-contrast micrographs of control and 24 h-EF40-treated cells. Arrested (at 25 μg/mL) and cytotoxic (at 50 μg/mL) phenotypes were observed in human NSCLC cells (A549 and H1299) but not in normal fibroblasts (MRC5 and TIG-3). **(B)** Cell viability assays of control and 24 h-EF40-treated cells showing a dose-dependent cytotoxicity in A549 and H1299 with a milder effect on the non-tumorigenic lung fibroblasts (MRC-5 and TIG-3). Statistical analysis between cancer and normal cells were performed using Two-way ANOVA multiple comparisons (^*a*^ *p* ≤ 0.001 vs. MRC5; ^*b*^ *p* ≤ 0.001 vs. TIG-3). **(C)** Long-term viability assay of control and EF40-treated cells showing a reduction in colony forming capacity in EF40-treated cells. Quantitation of colony numbers is shown on the right (mean ± SD, *n* = 3), ^***^ *p* ≤ 0.001 (Student’s t-test to Control). **(D)** EdU staining (EdU, green; Hoechst, blue) in A549 and H1299 cells showing a decrease in the number of EdU-positive proliferative cells after EF40 treatment for 24 h. Quantitation is shown on the right (mean ± SD, *n* = 3), ^*^ *p* ≤ 0.05), ^**^ *p* ≤ 0.01 (Student’s t-test to Control).

Next, we incubated the cells with EF40 for 24 h to observe morphological changes. As shown in Figure 1A, almost all NSCLC cells shrunk and appeared as round masses after 24 h of treatment with 50 μg/mL EF40, whereas TIG-3 and MRC5 normal cells were unaffected. Notably, NSCLC cells showed stronger cytotoxicity at all doses compared to normal cells (Figure 1B). Colonigenic assays with a low dose of EF40 (25 μg/mL) revealed a marked decrease in colony-forming efficiency (Figure 1C), suggesting that EF40 significantly inhibited the long-term growth of NSCLC cells. EdU staining was performed to determine DNA synthesis in cells in response to EF40 treatment. As expected, a reduced number of EdU-positive cells was observed in both A549 and H1299 cells following treatment. These findings prompted us to investigate the chemical composition of EF40. The UPLC-Orbitrap-MS analysis indicated the presence of 33 components (Supplementary Figure S3; Supplementary Table S1), among which 14 were reported to have anticancer activities (Supplementary Table S2). Summarily, these results indicate that EF40 is effective in NSCLC inhibition.

### EF40 causes ROS induction that is mediated by suppression of Nrf2-ARE signaling

**FIGURE 2:**
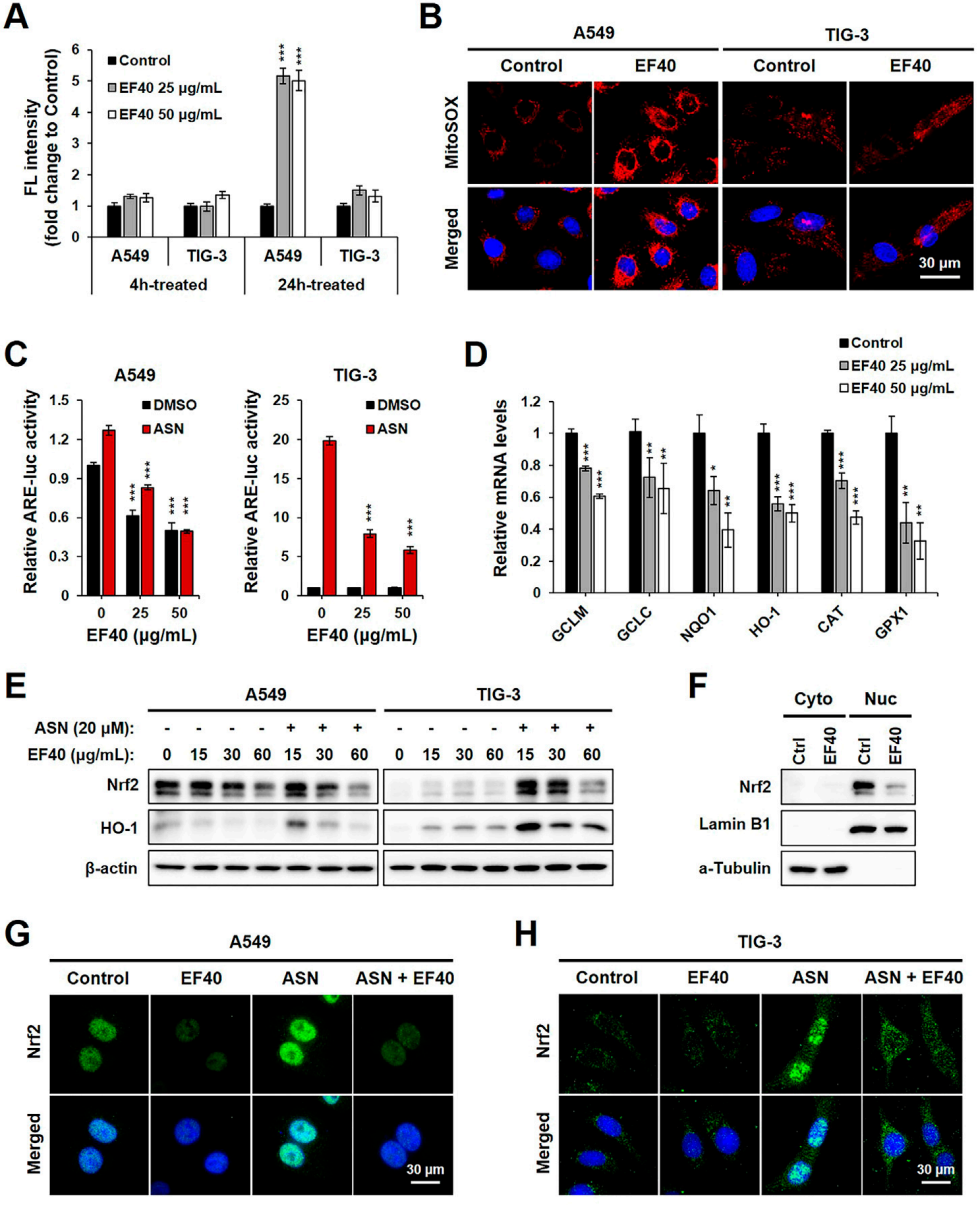
EF40 disrupts ROS homeostasis by suppression of NRF2-ARE signaling **(A)** Cellular ROS level measured by H_2_DCFDA assay showing that 24 h exposure to EF40 increases ROS in A549 but not in TIG-3 cells. Data are represented as mean ± SD (*n* = 3), ^***^ *p* ≤ 0.001 (Student’s t-test to Control). **(B)** Mitochondrial ROS level measured by MitoSOX staining showing elevated ROS production in EF40-treated (25 μg/mL, 24 h) A549 but not in TIG-3 cells. **(C)** ARE luciferase activity of control and EF40-treated cells in the presence or absence of sodium arsenite (ASN, 20 μM) for 16 h. Data are represented as mean ± SD (*n* = 3), ^***^ *p* ≤ 0.001 (Student’s t-test to 0 μg/mL). **(D)** qPCR analysis showing that 24 h treatment of EF40 reduced the expression levels of ARE target genes in A549 cells. Data are represented as mean ± SD (*n* = 3), ^*^ *p* ≤ 0.05, ^**^ *p* ≤ 0.01, ^***^ *p* ≤ 0.001 (Student’s t-test to Control). **(E)** Western blotting analyses showing decreased expressions of Nrf2 and HO-1 in EF40-treated A549 cells. ASN-induced Nrf2 and HO-1 in TIG-3 cells were also downregulated by EF40 treatment. Cells were treated with EF40 in combination with/without ASN (20 μM) for 6 h. **(F)** Western blotting analysis showing decreased nuclear level of Nrf2 in EF40-treated (25 μg/mL, 24 h) A549 cells. Lamin B1 and *α*-Tubulin were used as loading controls for nuclear and cytoplasm protein fractions, respectively. **(G)** Immunostaining of Nrf2 showing decreased expression in nuclei in EF40-treated (25 μg/mL) A549 cells with/without ASN induction (20 μM) for 6 h **(H)** TIG-3 cells showing cytoplasmic distribution of Nrf2 at low level; its induction by ASN (20 μM) was suppressed when co-incubated with EF40 (25 μg/mL) for 6 h.

To obtain molecular insights into the selective anticancer activity of EF40, its role in ROS production was examined. We observed that pulse treatment (4 h) with EF40 did not alter the ROS-driven fluorescence of H_2_DCFDA in A549 and TIG-3 cells. However, ROS production significantly increased in A549 cells when the treatment time was prolonged to 24 h. TIG-3 normal cells showed only a negligible increase following the same treatment (Figure 2A). These results suggest that the elevated ROS level was generated by disrupting the balance for cellular ROS production/elimination that occurred specifically in cancer cells. Hence, we examined intracellular ROS production in control and EF40-treated cells using MitoSOX, a mitochondria-specific ROS probe. The results showed a large increase in ROS levels following EF40 treatment (25 μg/mL, 24 h) in A549, but not TIG-3 cells (Figure 2B), suggesting the induction of mitochondrial ROS in cancer cells.

To investigate whether EF40-induced ROS production is involved in dysregulation of the Nrf2 signaling pathway, we examined ARE-dependent luciferase activity. ASN, an oxidative stressor, was used as a positive control. As shown in Figure 2C, EF40 suppressed the expression of ARE-luciferase in a concentration-dependent manner in A549 cells under both basal (DMSO) and ASN-challenged conditions. TIG-3 cells possessed low innate ARE activity, which was not altered by EF40. However, after ASN induction, ARE-driven luciferase was downregulated in EF40-treated TIG-3 cells, indicating that EF40 inhibits ARE promoter activity. We further confirmed the suppression of ARE activity by showing the attenuated mRNA expression of multiple ARE-targeted genes, including glutamate-cysteine ligase modifier (GCLM), glutamate-cysteine ligase catalytic subunit (GCLC), NAD(P)H quinone dehydrogenase 1 (NQO1), heme oxygenase 1 (HO-1), catalase (CAT), and glutathione peroxidase 1 (GPX1) (Figure 2D). In addition, Western blot analysis showed that EF40 depleted protein levels of Nrf2 and its downstream factor HO-1 in A549 cells under basal and ASN-treated conditions (Figure 2E, left panel). Interestingly, a slight increase in Nrf2 and HO-1 was observed in TIG-3 cells after EF40 treatment without ASN (Figure 2E, right panel), suggesting that EF40 causes cellular stress via signaling pathways independent of Nrf2. Furthermore, we found that EF40 hindered Nrf2 nuclear translocation (Figures 2F–H; Supplementary Figure S4), which in turn confirmed the repressed transcriptional activity of Nrf2-ARE signaling. Overall, these data demonstrate that the specific ROS induction by EF40 in NSCLC cells is likely due to its inhibitory effect on the Nrf2 pathway.

### Nuclear accumulation of ROS activates DNA damage in EF40-treated NSCLC cells

**FIGURE 3:**
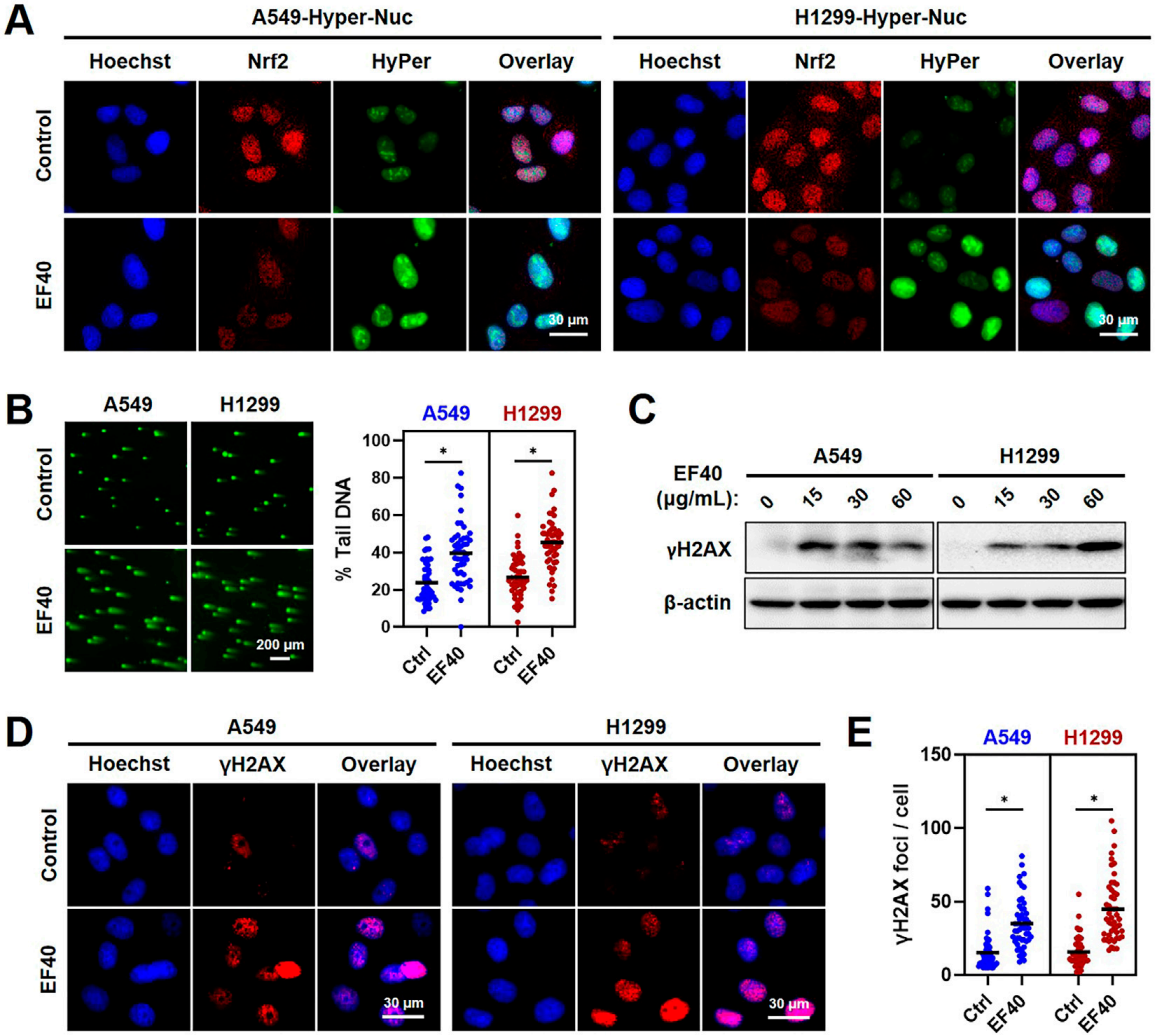
ROS accumulation resulted from EF40 treatment induces DNA damage in NSCLC cells **(A)** Fluorescence microscopy imaging of control and EF40-treated (25 μg/mL, 24 h) cells expressing HyPer-Nuc showing hydrogen peroxide induction in the nucleus along with decreased expression of Nrf2 in EF40-treated cells. **(B)** Comet assay in control and EF40-treated (25 μg/mL, 24 h) cells showing increase in DNA damage in EF40-treated cells. Quantitation from three independent experiments is shown on the right. ^*^ *p* ≤ 0.05 (Student’s t-test). A549 and H1299 cells treated with EF40 for 24 h were analyzed by **(C)** Western blotting and **(D)** immunostaining (EF40, 25 μg/mL) for the expression level of γH2AX with respect to the untreated control cells. An increase in γH2AX was observed in both methods. **(E)** γH2AX foci were quantified as foci per cell. Total of ∼100 cells were examined in each analysis. Black bars represent the mean values, ^*^ *p* ≤ 0.05 (Student’s t-test).

Given that increased ROS levels lead to genomic instability, we investigated whether EF40 triggers DNA damage in NSCLC cells. Hence, we first examined ROS accumulation in the nucleus using a fluorescent sensor (Hyper-Nuc) capable of detecting nuclear hydrogen peroxide. Fluorescence microscopy of A549 and H1299 cells following EF40 treatment revealed an increased level of nuclear ROS accompanied by decreased expression of Nrf2 (Figure 3A). Consequently, such ROS induction in the nucleus led to a marked increase in the formation of DNA double-strand breaks, as evidenced by the increased percentage of DNA in the tail of EF40-treated cells (Figure 3B). In accordance with this notion, Western blotting and immunofluorescence also revealed a profound increase in the expression level of γH2AX (an established DNA damage marker) in response to EF40 treatment (Figures 3C–E). Of note, pretreatment with Nrf2 inhibitor (ML-385) compromised EF40-induced DNA damage (Supplementary Figure S5) and alleviated the toxicity of EF40 in A549 cells (Supplementary Figure S6), suggesting the therapeutic effect of EF40 is dependent on Nrf2 inhibition. Collectively, these findings demonstrated that EF40-mediated Nrf2 inhibition induces nuclear ROS production and triggers DNA damage in NSCLC cells.

### EF40-triggered DNA damage induces cell cycle arrest and apoptosis in NSCLC cells

**FIGURE 4:**
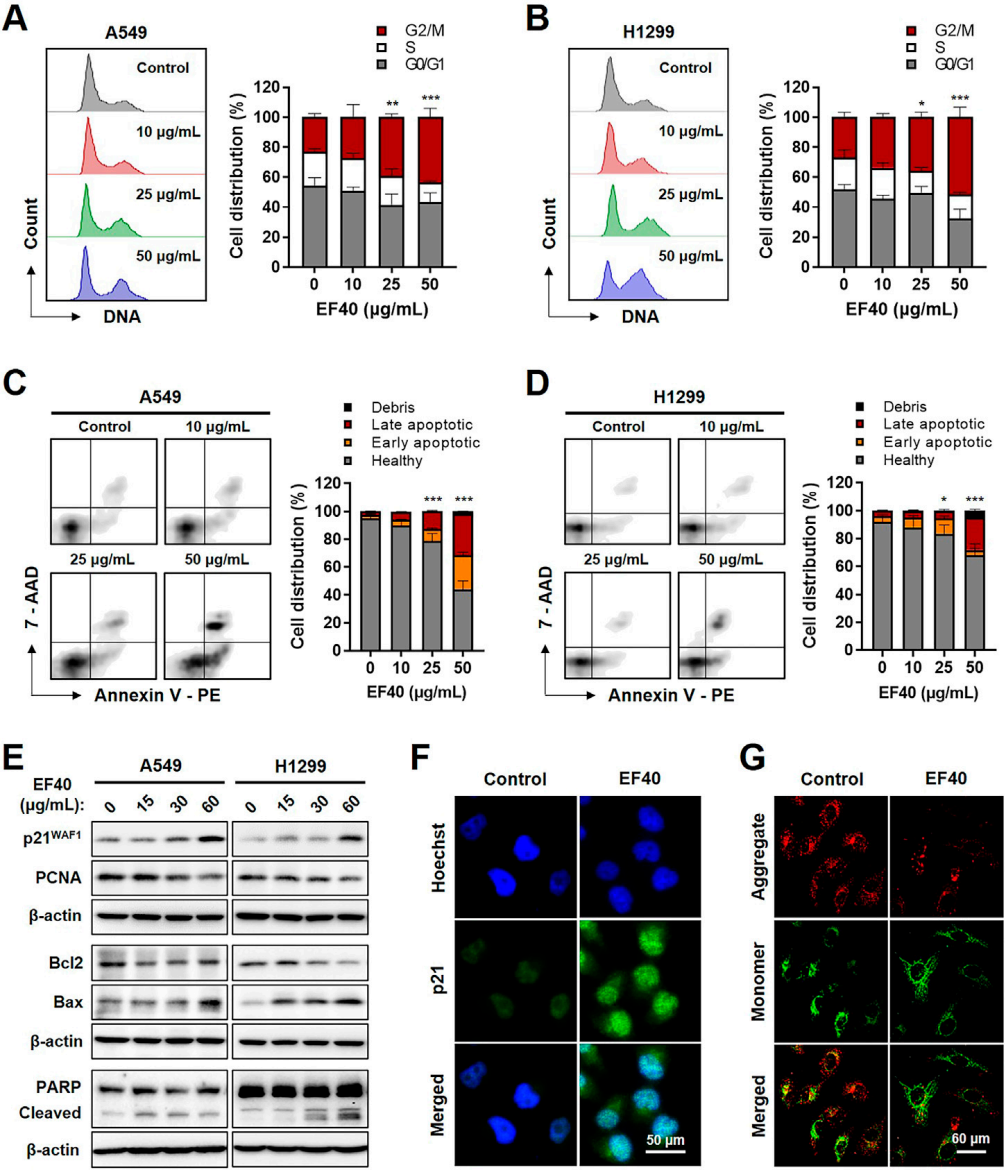
EF40-treated NSCLC cells undergo cell cycle arrest and apoptosis Cell cycle progression analyses for **(A)** A549 and **(B)** H1299 cells showing increase in cell population at G2/M arrest owing to EF40 treatment for 24 h. The quantified data are represented as mean ± SD (*n* = 3); significant differences in cell number of G2/M phase were identified, ^***^ *p* ≤ 0.001 (Two-way ANOVA test to 0 μg/mL). **(C, D)** Flow cytometric analysis showing a greater increase in apoptotic cell numbers with 24 h treatment of EF40 (50 μg/mL). The quantified data are represented as mean ± SD (*n* = 3); significant differences in cell number of apoptotic cells (early and late) were identified, ^***^ *p* ≤ 0.001 (Two-way ANOVA test to 0 μg/mL). **(E)** Western blotting for molecular markers of cell growth arrest and apoptosis. Treatment with EF40 for 24 h showing a dose-dependent increase in p21^WAF1^, Bax, and cleaved PARP. A decline in PCNA and Bcl2 was observed in EF40-treated cells. **(F)** Immunostaining of p21^WAF1^ showing increased expression level in EF40-treated (25 μg/mL, 24 h) A549 cells. **(G)** Analysis of mitochondrial membrane potential of A549 cells showing the depolarization after EF40 treatment (50 μg/mL, 24 h), as evident by the lack of red JC-1 aggregate accumulation.

To investigate the biological effects of EF40-induced DNA damage, we examined control and EF40-treated cells for growth arrest and apoptosis by flow cytometry. As shown in Figure 4A, cell cycle analysis indicated that EF40 caused G2/M arrest in the A549 cells. The cell population in the G2/M phase increased from 22.88% in the control cells to 24.31%, 26.74%, and 38.38% in cells treated with 10, 25, and 50 μg/mL EF40, respectively. Similar effects were observed in H1299 cells (Figure 4B). Furthermore, flow cytometric analysis after dual staining of cells with Annexin V-PE and 7-aminoactinomycin D (7-ADD) showed a higher extent of apoptosis in EF40-treated cells than in control cells in a dose-dependent manner (Figures 4C,D). This result is consistent with the typical apoptotic cell death morphology shown in Figure 1A. Quantitative results revealed that 53.6% and 27.13% of cells underwent apoptosis (early and late) after treatment with 50 μg/mL EF40 in A549 and H1299 cells, respectively (Figures 4C,D).

Next, we examined the expression of key regulatory proteins controlling cell cycle progression and apoptosis initiation in response to EF40 treatment. As shown in Figure 4E, Western blotting revealed a dose-dependent increase in p21^WAF1/CIP1^ expression and a decline in proliferating cell nuclear antigen (PCNA) expression in EF40-treated cells. Consistent with this phenotype, immunofluorescence also showed the upregulated expression of p21^WAF1/CIP1^ in A549 cells upon EF40 treatment (Figure 4F). Furthermore, a decrease in the anti-apoptotic protein B-Cell lymphoma-extra large (Bcl-xL) with an increase in proapoptotic proteins, BCL2-associated X (Bax) and cleaved poly-(ADP-ribose) polymerase (PARP), was found in the treated cells (Figure 4E), confirming the induction of apoptosis by EF40. As mitochondrial depolarization is the initial event in the apoptosis cascade, we analyzed the effect of EF40 on mitochondrial membrane potential. As expected, we observed reduced JC-1 aggregates in A549 cells following EF40 treatment (Figure 4G), suggesting that EF40 causes mitochondrial dysfunction. Taken together, these results clearly indicate that EF40 induces cell cycle arrest and apoptosis in NSCLC cells.

### EF40 inhibits migration and invasion activities of NSCLC cells

**FIGURE 5:**
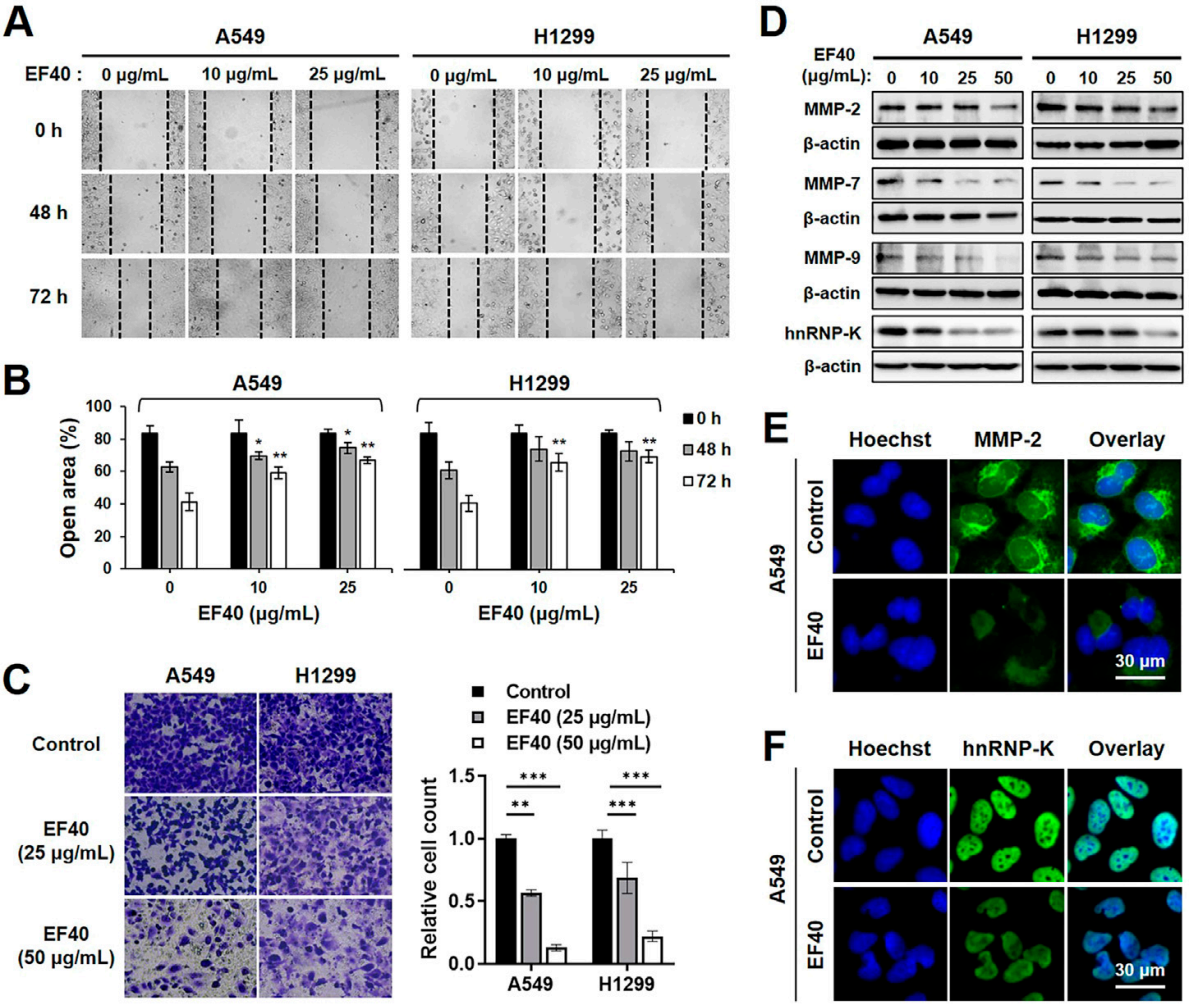
EF40 inhibits migration and invasion in NSCLC cells **(A)** Wound healing assay showing reduced cell migration in EF40-treated cells (magnification, ×200). **(B)** Quantitation of open area of the wound is shown (mean ± SD; *n* = 3). ^*^ *p* ≤ 0.05, ^**^ *p* ≤ 0.01 (one-way ANOVA test to 0 μg/mL). **(C)** Invasion assays and its quantitation showing strong inhibition with 6 h treatment of EF40 (magnification, ×100). Quantitative data are represented as mean ± SD (*n* = 3). ^**^ *p* ≤ 0.01, ^***^ *p* ≤ 0.001 (one-way ANOVA test). **(D)** Western blotting and **(E, F)** immunostaining analyses of MMPs and hnRNP-K proteins showing decreased expression levels after EF40 treatment. A549 cells were treated with 25 μg/mL of EF40 for 24 h in **(E, F)**.

As NSCLC cells are highly metastatic, we investigated the effect of EF40 on cell migration. The Wound scratch assay of A549 and H1299 cells revealed that EF40 significantly delayed the motility of both cells (Figures 5A,B). The cell invasion capacity was also evaluated in control cells and those treated with EF40. As shown in Figure 5C, a clear reduction in the number of invasive cells was observed after treatment. Quantitation of results from three independent experiments revealed that the invasiveness of A549 and H1299 cells decreased to 13% and 22%, respectively, after EF40 treatment (50 μg/mL) for 6 h. These data suggest that EF40 inhibits NSCLC metastasis *in vitro*.

Matrix metalloproteinases (MMPs) play a crucial role in tumor metastasis by degrading extracellular matrix proteins (Choe et al., 2002). To identify the possible alterations in MMPs in NSCLC cells caused by EF40, the expression levels of MMP-2, MMP-7, and MMP-9 were assessed by Western blotting. As shown in Figure 5D, MMP-2 expression in A549 and H1299 cells was downregulated in the presence of EF40 at 50 μg/mL. These results were also supported by immunofluorescence data with a lower dose of EF40 at 25 μg/mL (Figure 5E). Meanwhile, a sharp decrease in MMP-7 and MMP-9 levels was observed after EF40 treatment in a dose-dependent manner in both cell types (Figure 5D). Heterogeneous nuclear ribonucleoprotein K (hnRNP-K) acts as a prognostic biomarker for metastasis, positively regulating MMP expression (Chen et al., 2010; Gao et al., 2013). Western blotting and immunofluorescence analysis of hnRNP-K revealed a significant decrease in its expression following EF40 treatment (Figures 5D,F). These results further confirmed the ability of EF40 to inhibit cancer metastasis that is mediated by repression of the hnRNP-K-MMP axis.

### EF40 impedes the growth and metastasis of NSCLC cells *in vivo*

**FIGURE 6:**
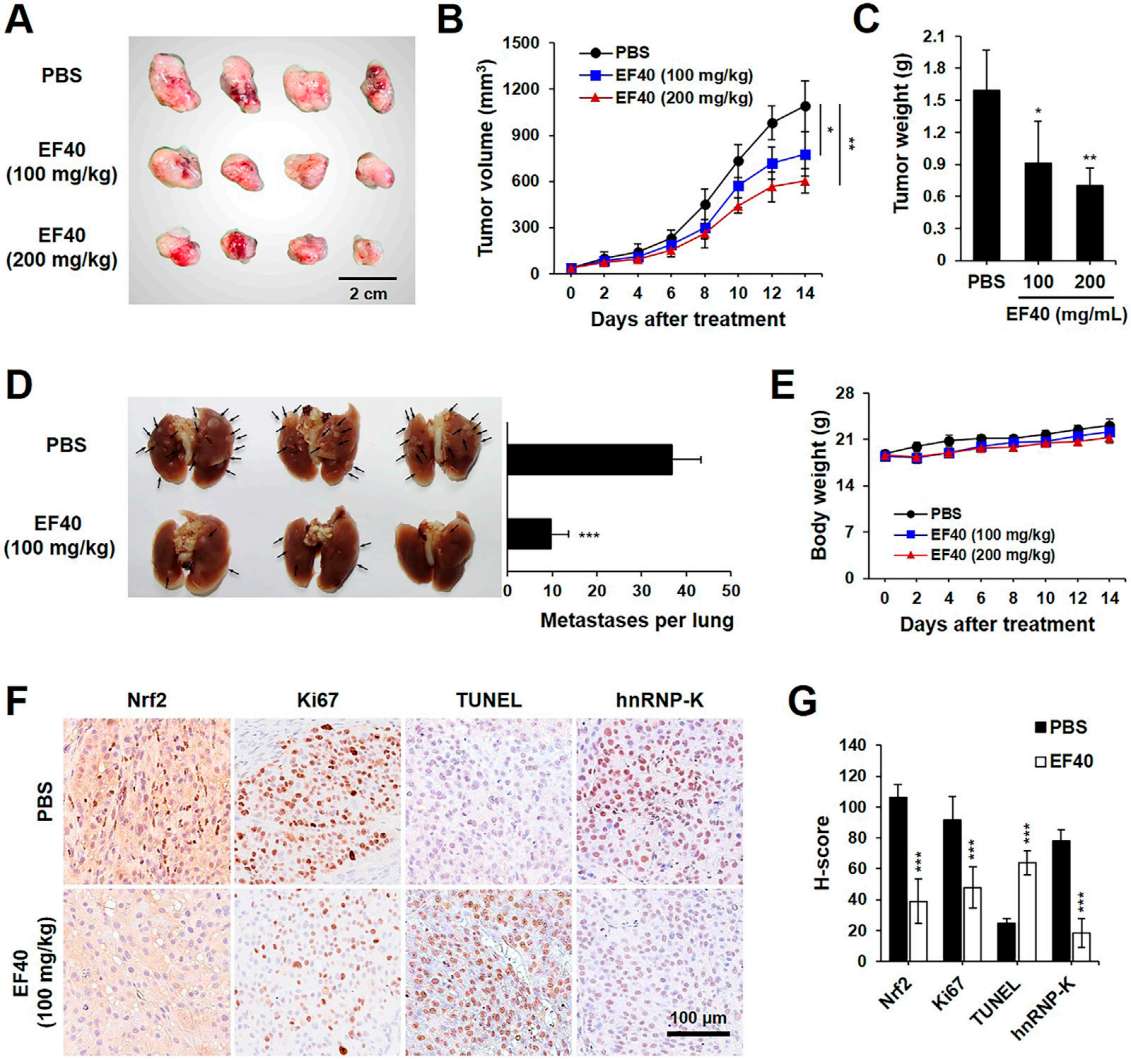
EF40 suppresses tumor growth and metastasis in NSCLC xenografts **(A)** Gross image of subcutaneous xenografts derived from A549 cancer cells with intraperitoneal injection of PBS and EF40. **(B)** Tumor volumes in different groups of mice during the course of treatment (mean ± SD; *n* = 4), ^*^ *p* ≤ 0.05, ^**^ *p* ≤ 0.01 (two-way ANOVA test). Subcutaneous xenografts in nude mice treated with EF40 showed retarded tumor growth rate. **(C)** Weight of xenograft tumors at the end of the treatments (mean ± SD; *n* = 4), ^*^ *p* ≤ 0.05, ^**^ *p* ≤ 0.01 (Student's t-test to PBS). **(D)** Image of lung metastases showing the suppression of lung tumors in EF40-treated (oral administration) mice as compared with PBS control. Quantitative result of metastases per lung is shown on the right (mean ± SD; *n* = 3), ^***^ *p* ≤ 0.001 (Student's t-test to PBS). **(E)** Average body weight of mice showing no significant loss during treatments. **(F)** Immunohistochemical analysis showing downregulations of Nrf2, Ki-67, and hnRNP-K and upregulation of TUNEL in tumor tissues harvested from mice treated with EF40. **(G)** Their quantitative H-scores are shown on the right (mean ± SD; *n* = 4), ^***^ *p* ≤ 0.001 (Student's t-test to PBS).

The data presented above warranted an *in vivo* assessment of the anti-cancer effects of EF40. A549 tumor-bearing BALB/c nude mice were randomly allocated into control and EF40 treatment groups after the tumors reached the desired size (ca. 35 mm^3^). The mice in the treatment group were administered EF40 at 100 or 200 mg/kg body weight daily, whereas the mice in the vehicle control group were administered PBS solution. After administration for 2 weeks, both tumor volume and weight were reduced in EF40-treated mice (Figures 6A–C), indicating the inhibitory effects of EF40 on tumor growth. The antimetastatic activity of EF40 in nude mice was investigated using a pulmonary metastasis assay. As shown in Figure 6D, control mice showed large lung metastatic tumors with an average number of ca. 36.8, whereas EF40-treated mice showed significant reductions in both size and number of lung metastases (ca. 9.8), suggesting an encouraging anti-metastatic activity. Notably, no evident toxicity was observed during the experiment in the treated animals when comparing the body weight changes with the control group animals (Figure 6E), demonstrating that EF40 has low systemic toxicity.

Immunohistochemistry staining was performed to obtain molecular evidence for the regulation of tumor growth and metastasis by EF40. Compared to the untreated counterpart (PBS group), EF40 treatment led to a substantial decrease in the expression levels of Nrf2, Ki67 (proliferation marker), and hnRNP-K and upregulated the expression of TUNEL (apoptosis marker) (Figures 6F,G). These results are highly consistent with our *in vitro* observations and suggest that EF40 retards tumor growth and metastasis of NSCLC cells by targeting Nrf2 and hnRNP-K, respectively.

## Discussion

Although massive efforts have been made, the overall survival of patients with NSCLC have not yet improved. The modulation of intracellular oxidative stress is considered an effective approach for cancer treatment. Our previous study found that AQHAR induced ROS induction and DNA damage in human cancer cells (Li et al., 2016a), and its major cytotoxic factor, EF, exhibited remarkable cytotoxicity against the NSCLC cell lines A549 and H1299 (Li et al., 2016b). However, the underlying molecular mechanism remains unclear. In the present study, EF40 was isolated from EF, and its effects on the proliferation and migration of NSCLC cells were evaluated. These results suggest that EF40 decreased the proliferation of A549 and H1299 cells by inhibiting Nrf2 activity. The transcription factor Nrf2 regulates the expression of antioxidant genes and protects cells against a plethora of environmentally or endogenously deleterious attacks. However, the cytoprotective role of Nrf2 is destructive for cancers because it confers a survival advantage to cancer cells along with resistance to chemo- and radiotherapy (Robledinos-Antón et al., 2019). Dysregulation of Nrf2 is frequently found in many types of lung cancers. Emerging evidence has shown that overactivation of the Nrf2 signaling pathway is associated with poor prognosis in NSCLC (Hammad et al., 2019; Zhao et al., 2020). Therefore, it is reasonable to assume that Nrf2 inhibition could improve the outcomes of NSCLC therapy. Herein, we report that EF40 is a good candidate for NSCLC therapy. It downregulated Nrf2 expression in cancer cells (Figure 2E) and xenografts (Figure 6F) at a dose that selectively killed NSCLC cells without affecting normal lung fibroblasts (Figure 1A). We confirmed that EF40-mediated inhibition of Nrf2 caused transcriptional repression of its downstream defensive genes (Figure 2D), resulting in mitochondrial oxidative stress and dysfunction (Figure 2B; Figure 4G).

Excessive ROS production may cause irreparable oxidative damage to DNA, proteins, and lipids, resulting to cell death (Piccirillo et al., 2009). Fluorescence imaging of cells expressing the hydrogen peroxide indicator showed nuclear accumulation of ROS after EF40 treatment (Figure 3A), which subsequently activates the DNA damage response in NSCLC cells (Figures 3B–D). Cancer cells can proliferate without limit. One of the most important features of an effective anticancer drug is the control of cancer cell progression and induction of apoptosis as required (Abedin and Barua, 2021). Extensive biochemical analysis revealed that EF40 arrests NSLCL cells in the G2/M phase in response to ROS-induced DNA damage and ultimately triggers apoptosis, probably due to the failure of DNA repair (Figure 4). Our results demonstrated the potential therapeutic use of EF40 to combat NSCLC.

Cancer cell migration and invasion are critical steps in tumor metastasis. Suppression of this process may improve therapeutic outcomes (Deng et al., 2016). MMPs are upregulated in almost every type of cancer and are regarded as primary factors in cancer development, including proliferation, invasion, and metastasis (Merdad et al., 2014). Among the 23 members of the MMP family, MMP-2, MMP7, and MMP-9 are regarded as key factors involved in cancer metastasis (Eiseler et al., 2009; Shi et al., 2018). In the present study, the effects of EF40 on MMP expression were identified by Western blotting and immunostaining in A549 and H1299 cells. As illustrated in Figure 5, treatment with EF40 substantially suppressed the expression of MMP-2, MMP-7, and MMP-9, indicating that EF40 regulated NSCLC cell migration and invasion by modulating MMP expression. These data were also supported by the decreased expression of hnRNP-K, an upstream activator of MMPs (Chen et al., 2010; Gao et al., 2013).

**FIGURE 7:**
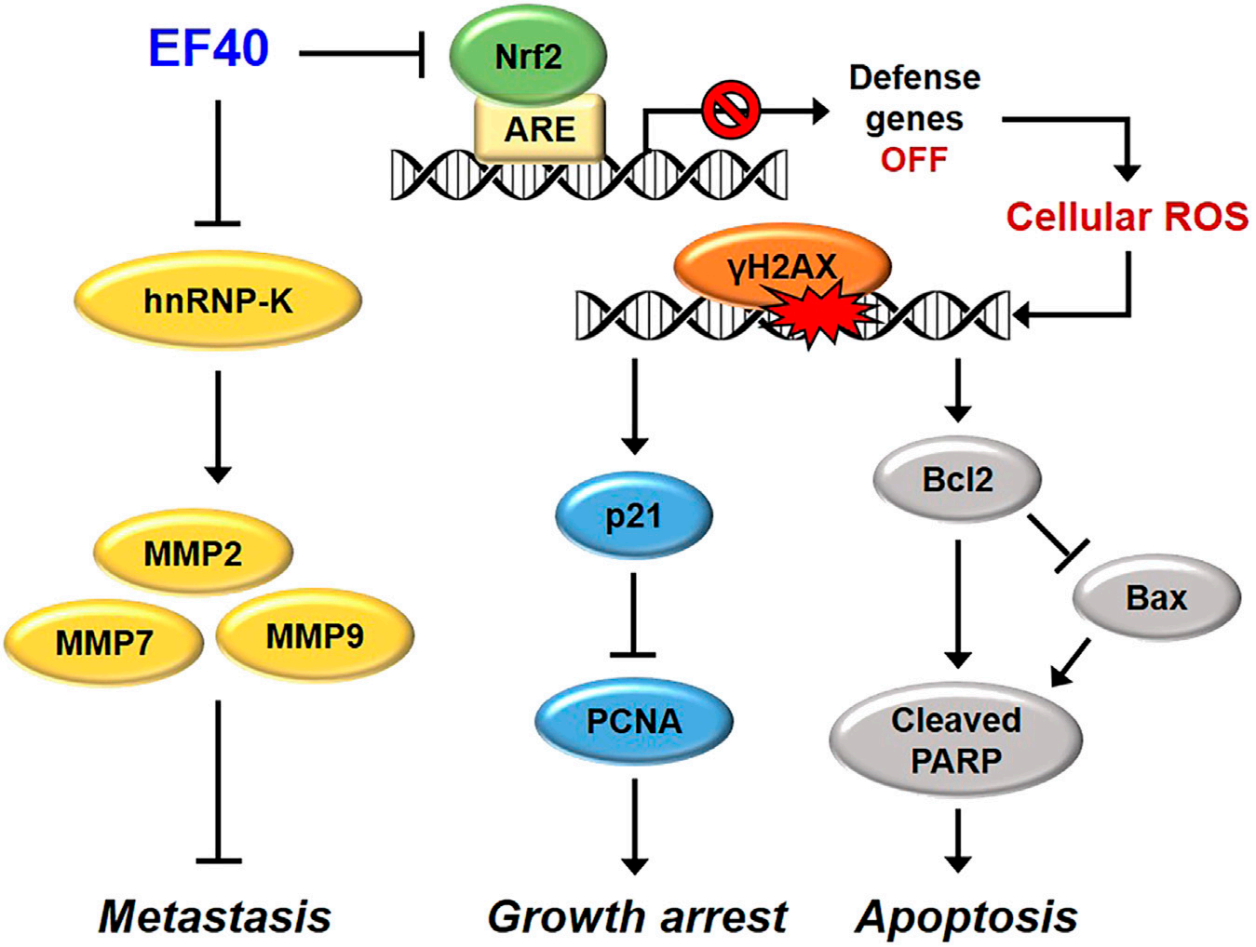
Schematic model showing the molecular mechanisms of anticancer effects of EF40. EF40 downregulates hnRNP-K, resulting in MMP-mediated repression of metastasis. The aberrantly activated Nrf2 in NSCLC cells is targeted by EF40, leading to transcriptional inhibition of ARE-dependent genes, thereby elevating intracellular ROS level. Such ROS induction, in turn, triggers DNA damage-induced growth arrest and apoptosis.

Based on the above data, we investigated the antitumor and antimetastatic activities of EF40 using xenograft mouse models of metastatic A549 tumors. EF40 administration remarkably retarded A549 growth and tumor metastasis, with inhibition rates of nearly 42.71% and 73.37%, respectively (Figures 6A–D). At the molecular level, EF40 downregulated the expression of Nrf2, Ki67, and hnRNP-K and upregulated TUNEL expression in tumor tissues. Our previous study demonstrated that AQHAR (400 mg/mL) caused 55.46% reduction in lung metastasis (Li et al., 2016a). The therapeutic efficacy of EF40 identified in this study was superior to that of AQHAR, indicating that EF40 is a better drug candidate. In summary, our results demonstrate that EF40 targets Nrf2, leading to the transcriptional inhibition of ARE-dependent genes, thereby elevating intracellular ROS levels. This ROS induction, in turn, triggers DNA damage-induced growth arrest and apoptosis. Moreover, EF40 downregulated hnRNP-K, resulting in MMP-mediated repression of metastasis in NSCLC cells (Figure 7). It is worth noting that ARE-like sequences were found in the proximal region of Nrf2 promoter. Therefore, the expression of Nrf2 could be self-regulated through direct binding to its own promoter (Kwak et al., 2002). One possible mechanism for EF40-induced reduction of Nrf2 is that downregulated Nrf2 protein may attenuate its binding to ARE-like elements located on Nrf2 promoter, and thus leads to transcriptional repression. However, the detailed mechanism by which EF40 enhances Nrf2 inhibition warrants more investigations. Moreover, to reduce administration amount of EF40 for better clinical translation, continued extensive studies are highly required to identify and isolate effective chemical compounds in the EF40 extract, success of which may further highlight the value of EF40 as an effective anticancer drug.

## Data Availability

All data supporting the findings of this study are available within the article and the Supplementary materials and have been deposited in the publicly accessible figshare repository: http://doi.org/10.6084/m9.figshare.23358032 The raw data can be obtained by running the source data files and can also be made available by the corresponding author upon reasonable request.

## Abbreviations

AQHAR, aqueous extract of *H. angustifolia* root; AREs, antioxidant response elements; ASN, sodium arsenite; EF, Ethanol fraction of AQHAR; EF40, 40% ethanol fraction of EF; *H. angustifolia*, *Helicteres angustifolia* L; MMPs, Matrix Metalloproteinases; MTT, 3-(4, 5- dimethylthiazol-2-yl)-2, 5-diphenyltetrazolium bromide; NSCLC, non-small cell lung cancer; Nrf2, Nuclear factor-E2-related factor 2; ROS, Reactive oxygen species; WF, water fraction of AQHAR.
