# Supplementary material for: Bioactivity-guided fractionation of Helicteres angustifolia L. extract and its molecular evidence for tumor suppression
Source: Front Cell Dev Biol. 2023 Jun 22;11:1157172. doi: 10.3389/fcell.2023.1157172 (PMC10323433; doi:10.3389/fcell.2023.1157172)
Supplement: Supplementary file 1 [file DataSheet1.docx]

Supplementary Material

**Bioactivity-guided fractionation of *Helicteres angustifolia L.* extract and its molecular evidence for tumor suppression**

**Kejuan Li, Shuang Sun, Long Xiao and Zhenya Zhang**

***Correspondence:** [likejuan@sicnu.edu.cn](mailto:likejuan@sicnu.edu.cn) (K. Li)

**
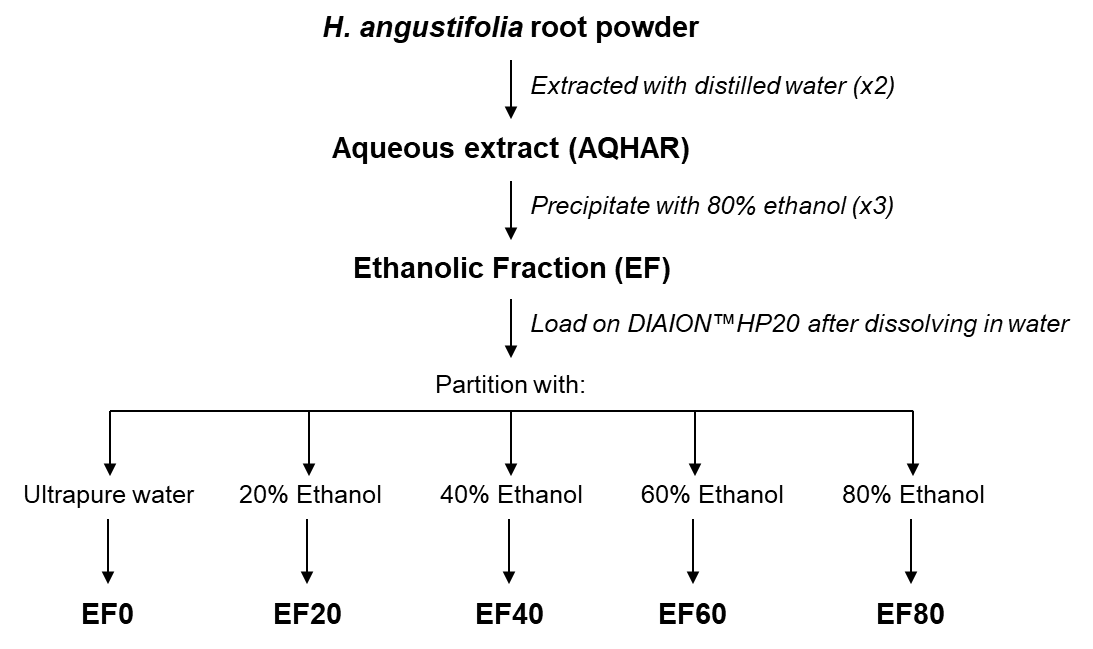
**

**Supplementary Figure 1.** Extraction method of ethanolic fractions from *H. angustifolia* root


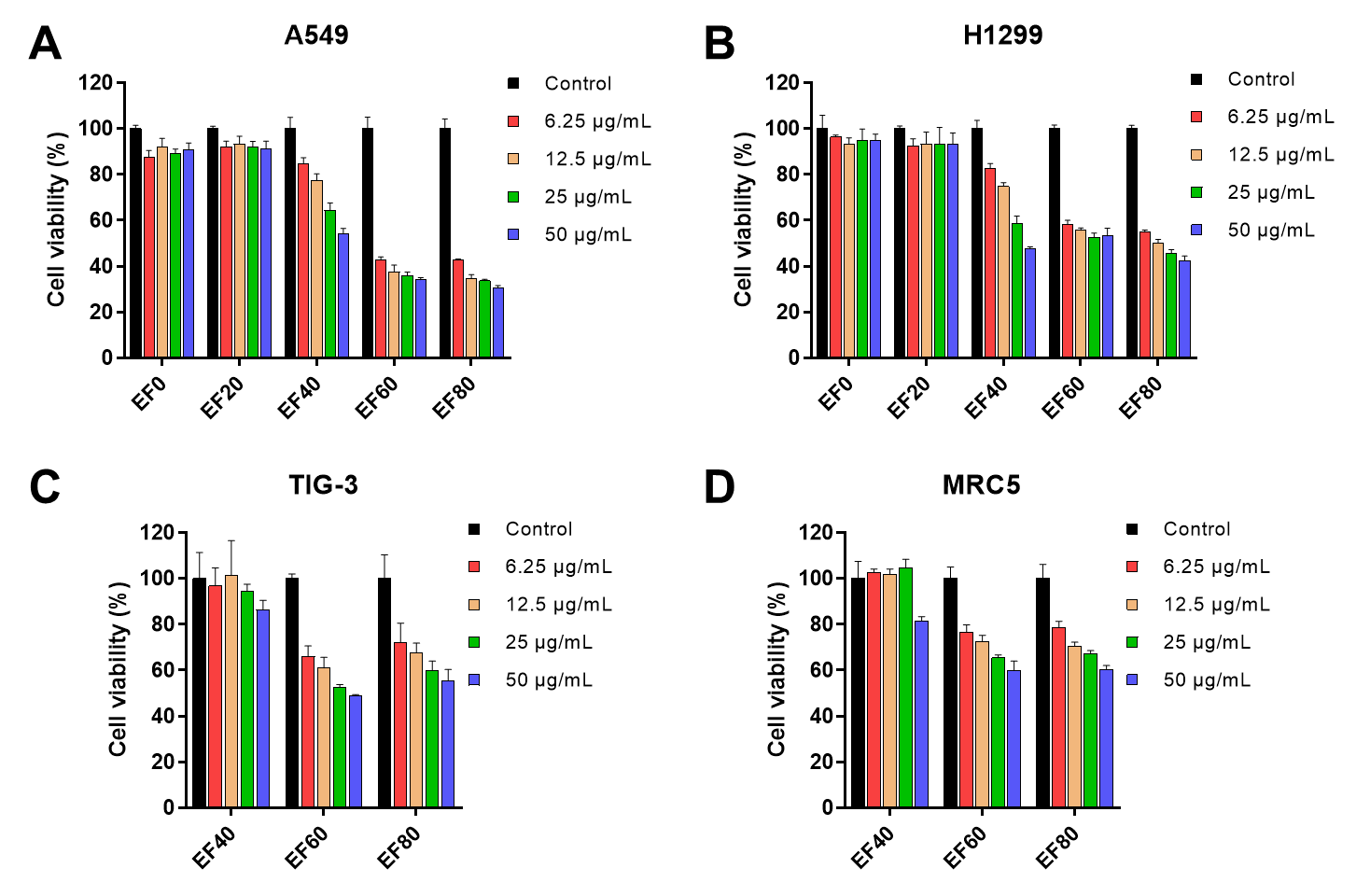


**Supplementary Figure 2.** Cell viability assays of NSCLC cells (**A** and **B**) and normal lung fibroblasts (**C** and **D**) with 24 h treatment with ethanolic fractions of *H. angustifolia* root.


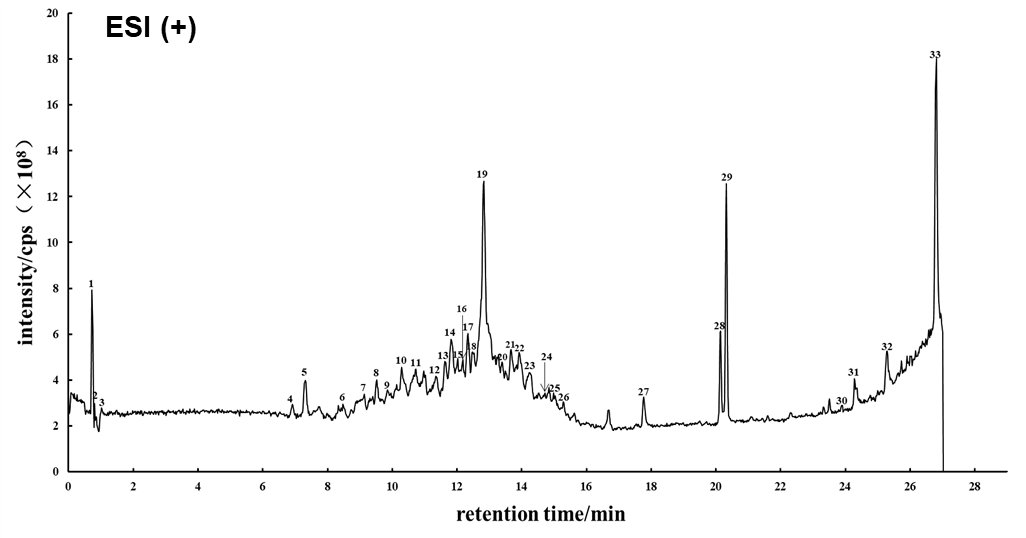


**Supplementary Figure 3.** LC-MS chromatographic fingerprint analysis of the EF40.


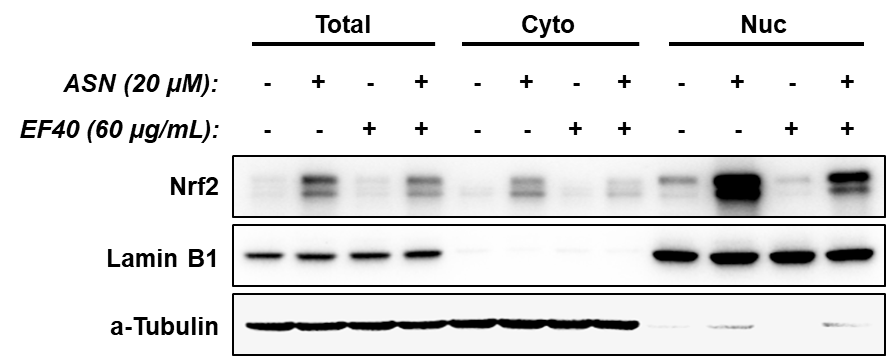


**Supplementary Figure 4.** EF40 reduced protein level of Nrf2 under ASN-challenged condition in the whole cell lysate and fractions of cytoplasm and nucleus. TIG-3 cells were treated with EF40 in the presence or absence of ASN for 8 h.


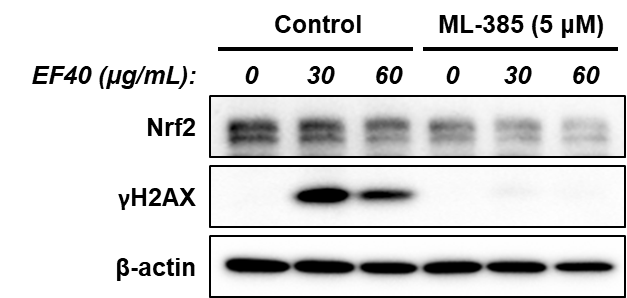


**Supplementary Figure 5.** Nrf2 repression by ML-385 inhibitor compromised EF40-induced DNA damage. A549 cells were left untreated or treated with 5 μM of ML-385 for 24h, followed by EF40 treatment for additional 24 h.


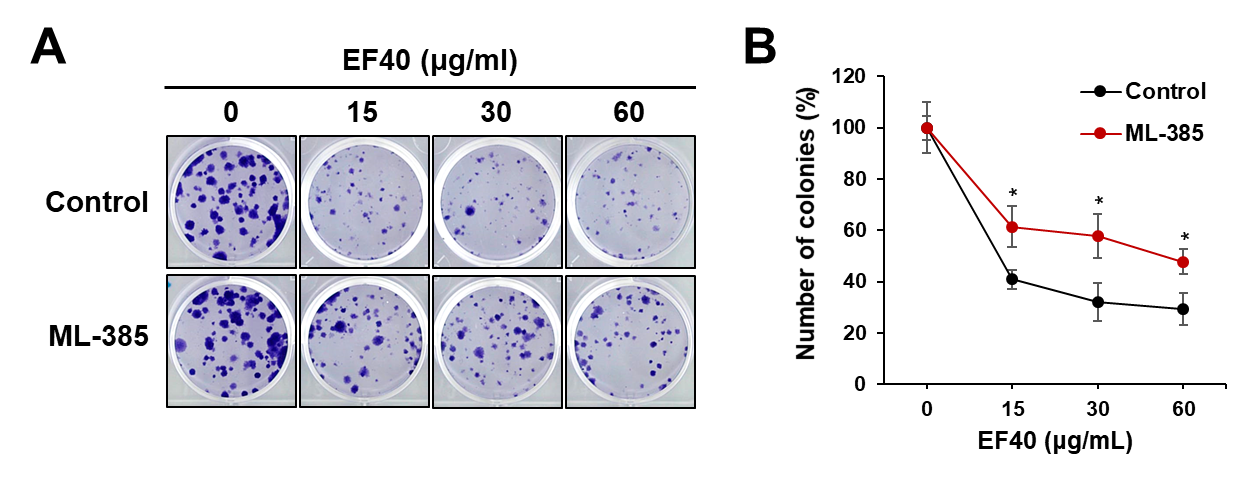


**Supplementary Figure 6.** (**A**) Pretreatment with ML-385 (a Nrf2 inhibitor) at 5 μM for 24 h compromised EF40-induced reduction in colony forming ability of A549 cells. (**B**) Quantitation of colony numbers is shown (mean ± SD, n = 3), *P ≤ 0.05 (Student’s t-test to Control).

**Supplementary Table 1.** Identification of 33 chemical components in EF40

| **Peak No.** | **Retention Time/min** | **Molecular Weight** | **Molecular Formula** | **Inferred Compounds** |
| --- | --- | --- | --- | --- |
| 1 | 0.726 | 176.13 | C_5_H_8_N_2_O_5_ | 3-[(Carboxycarbonyl)amino]-L-alanine |
| 2 | 0.803 | 198.15 | C_9_H_18_N_4_O | Amidinomycin |
| 3 | 1.020 | 342.12 | C_12_H_22_O_11_ | α,α-Trehalose |
| 4 | 6.911 | 206.06 | C_11_H_10_O_4_ | Scoparone |
| 5 | 7.309 | 350.01 | C_15_H_10_O_8_S | Apigenin 7-sulfate |
| 6 | 8.476 | 258.09 | C_15_H_14_O_4_ | Isorhapontigenin |
| 7 | 9.137 | 574.31 | C_32_H_46_O_9_ | Cucurbitacin A |
| 8 | 9.525 | 164.08 | C_10_H_12_O_2_ | Eugenol |
| 9 | 9.851 | 376.14 | C_17_H_20_N_4_O_6_ | Riboflavin |
| 10 | 10.292 | 464.29 | C_30_H_40_O_4_ | Pristimerin |
| 11 | 10.733 | 394.13 | C_19_H_22_O_9_ | Aloesin |
| 12 | 11.345 | 556.30 | C_32_H_44_O_8_ | Cucurbitacin E |
| 13 | 11.631 | 448.17 | C_23_H_28_O_9_ | 2,3,4,5,2',3',4',6'-Octamethoxychalcone |
| 14 | 11.822 | 718.15 | C_36_H_30_O_16_ | Salvianolicacid B |
| 15 | 12.031 | 518.32 | C_30_H_46_O_7_ | Cucurbitacin F |
| 16 | 12.183 | 314.08 | C_18_H_16_O_8_ | Rosmarinic acid |
| 17 | 12.336 | 274.08 | C_15_H_14_O_5_ | Phloretin |
| 18 | 12.489 | 180.04 | C_9_H_8_O_4_ | Caffeic acid |
| 19 | 12.831 | 280.17 | C_16_H_24_O_4_ | (+)-brefeldin A |
| 20 | 13.383 | 280.13 | C_15_H_20_O_5_ | 8'-Hydroxyabscisate |
| 21 | 13.669 | 300.21 | C_20_H_28_O_2_ | 9-cis-Retinoicacid |
| 22 | 13.917 | 622.19 | C_29_H_34_O_15_ | 6,8-Dihydroxy-7,4'-dimethoxyflavone 6-rutinoside |
| 23 | 14.223 | 788.38 | C_38_H_60_O_17_ | Dulcoside A |
| 24 | 14.721 | 186.03 | C_11_H_6_O_3_ | Angelicin |
| 25 | 14.99 | 246.09 | C_14_H_14_O_4_ | Columbianetin |
| 26 | 15.280 | 593.34 | C_36_H_43_N_5_O_3_ | 3-(2-Naphthyl)-N-(4-piperidinylcarbonyl)-D-alanyl-N-(4-aminobutyl)-3-(2-naphthyl)-D-alaninamide |
| 27 | 17.772 | 257.24 | C_15_H_31_NO_2_ | Lauric isopropanolamide |
| 28 | 20.146 | 257.20 | C_14_H_27_NO_3_ | N-lauroylglycine |
| 29 | 20.322 | 558.32 | C_32_H_46_O_8_ | Cucurbitacin B |
| 30 | 23.891 | 255.26 | C_16_H_33_NO | Hexadecanamide |
| 31 | 24.281 | 281.27 | C_18_H_35_NO | Oleamide |
| 32 | 25.287 | 383.34 | C_23_H_45_NO_3_ | Serinolamide A |
| 33 | 26.808 | 294.18 | C_17_H_26_O_4_ | 6-Gingerol |

**Supplementary Table 2** Classification of compounds identified form EF40. Compounds possessing anticancer activities according to the earlier studies are highlighted in blue.

| **Classifications** | **Compounds** |
| --- | --- |
| Triterpenes | Cucurbitacin A (Liu et al., 2018) |
|  | Pristimerin (Chen et al., 2021) |
|  | Cucurbitacin E (Yang et al., 2020) |
|  | Cucurbitacin F |
|  | Cucurbitacin B (Wang et al., 2021) |
|  | 8'-Hydroxyabscisate |
| Phenolics | Eugenol (Abdullah et al., 2021) |
|  | Salvianolicacid B (Wang et al., 2021) |
|  | Rosmarinic acid (Zhou et al., 2022) |
|  | Caffeic acid |
|  | [6]-Gingerol (Sp et al., 2021) |
| Coumarins | Scoparone (Li et al., 2021) |
|  | Angelicin (Mahendra et al., 2020) |
|  | Columbianetin |
| Flavonoids | Apigenin 7-sulfate |
|  | 2,3,4,5,2',3',4',6'-Octamethoxychalcone |
|  | Phloretin (Kim et al., 2020) |
|  | 6,8-Dihydroxy-7,4'-dimethoxyflavone 6-rutinoside |
| Stilbenes | Isorhapontigenin (Zhang et al., 2021) |
| Naphthalenones | Aloesin (Sánchez et al., 2020) |
| Lactones | (+)-brefeldin A (Zhang et al., 2021) |
| Amides | 3-(2-Naphthyl)-N-(4-piperidinylcarbonyl)-D-alanyl-N-(4-aminobutyl)-3-(2-naphthyl)-D-alaninamide |
|  | Lauric isopropanolamide |
|  | Hexadecanamide |
|  | Oleamide |
|  | Serinolamide A |
| Glycosides | Dulcoside A |
| Amino acids | N-lauroylglycine |
|  | 3-[(Carboxycarbonyl)amino]-L-alanine |
|  | Riboflavin |
|  | 9-cis-Retinoicacid |
| Aisaccharides | α,α-Trehalose |
| Antibiotics | Amidinomycin |

**Supplementary Table 3** Antibodies used in this study.

| **Antibody** | **Type** | **Label** | **Source** | **Catalog No.** | **Application** |
| --- | --- | --- | --- | --- | --- |
| Nrf2 | Rabbit | / | Santa Cruz | sc-13032 | WB (1:1000) |
|  | Polyclonal |  |  |  |  |
| Nrf2 | Rabbit Monoclonal | / | Abcam | ab62352 | WB (1:1000) |
|  |  |  |  |  | IF (1:500) |
|  |  |  |  |  | IHC (1:200) |
| HO-1 | Rabbit | / | Abcam | ab52947 | WB (1:2000) |
|  | Polyclonal |  |  |  |  |
| γH2AX | Rabbit | / | Cell Signaling | 9718 | WB (1:1000) |
|  | Monoclonal |  |  |  | IF (1:500) |
| p21^WAF1^ | Rabbit | / | Cell Signaling | 2947 | WB (1:1000) |
|  | Monoclonal |  |  |  | IF (1:500) |
| PCNA | Mouse | / | Abcam | ab29 | WB (1:3000) |
|  | Monoclonal |  |  |  |  |
| Bcl2 | Mouse | / | Santa Cruz | sc-7382 | WB (1:1000) |
|  | Monoclonal |  |  |  |  |
| Bax | Mouse | / | Santa Cruz | sc-7480 | WB (1:1000) |
|  | Monoclonal |  |  |  |  |
| PARP | Rabbit | / | Cell Signaling | 9542 | WB (1:1000) |
|  | Polyclonal |  |  |  |  |
| hnRNP-K | Rabbit | / | Cell Signaling | 4675 | WB (1:1000) |
|  | Polyclonal |  |  |  | IF (1:500) |
| hnRNP-K | Rabbit | / | Abcam | ab52600 | IHC (1:2000) |
|  | Monoclonal |  |  |  |  |
| MMP-2 | Mouse | / | Santa Cruz | sc-13594 | WB (1:1000) |
|  | Monoclonal |  |  |  | IF (1:250) |
| MMP-7 | Rabbit | / | Santa Cruz | sc-30071 | WB (1:1000) |
|  | Polyclonal |  |  |  |  |
| MMP-9 | Rabbit | / | Cell Signaling | 2270 | WB (1:1000) |
|  | Polyclonal |  |  |  |  |
| Ki-67 | Rabbit | / | Abcam | ab15580 | IHC (1:2000) |
|  | Polyclonal |  |  |  |  |
| Lamin B1 | Rabbit | / | Abcam | ab16048 | WB (1:2000) |
|  | Polyclonal |  |  |  |  |
| α-Tubulin | Rabbit | / | Abcam | ab4074 | WB (1:2000) |
|  | Polyclonal |  |  |  |  |
| β-actin | Mouse | HRP | Abcam | ab49900 | WB (1:30000) |
|  | Monoclonal |  |  |  |  |
| Anti-Rabbit | Goat | HRP | Cell Signaling | 7074 | WB (1:5000) |
|  | Polyclonal |  |  |  |  |
| Anti-Mouse | Horse | HRP | Cell Signaling | 7076 | WB (1:5000) |
|  | Polyclonal |  |  |  |  |
| Anti-Mouse | Goat | Alexa Fluor 488 | Thermo Fisher | A-11001 | IF (1:500) |
|  | Polyclonal |  |  |  |  |
| Anti-Rabbit | Goat | Alexa Fluor 488 | Thermo Fisher | A-11008 | IF (1:500) |
|  | Polyclonal |  |  |  |  |
| Anti-Rabbit | Goat | Alexa Fluor 594 | Thermo Fisher | A-11012 | IF (1:500) |
|  | Polyclonal |  |  |  |  |

**Supplementary Table 4** Primers used in RT-qPCR.

| **Gene** | **Forward primer (5’→3’)** | **Reverse primer (5’→3’)** |
| --- | --- | --- |
| hGCLM | TGTCTTGGAATGCACTGTATCTC | CCCAGTAAGGCTGTAAATGCTC |
| hGCLC | AGAGAAGGGGGAAAGGACAAAC | AAGTTATTGTGCAAAGAGCCTGAT |
| hNQO1 | ATGTATGACAAAGGACCCTTCC | TCCCTTGCAGAGAGTACATGG |
| hHO-1 | AACTTTCAGAAGGGCCAGGT | CTGGGCTCTCCTTGTTGC |
| hCAT | ACTTTGAGGTCACACATGACATT | CTGAACCCGATTCTCCAGCA |
| hGPX1 | TGCAACCAGTTTGGGCATCA | ACCGTTCACCTCGCACTTC |
| 18S | CAGGGTTCGATTCCGTAGAG | CCTCCAGTGGATCCTCGTTA |

**References**

Abdullah M.L., Al-Shabanah O., Hassan Z.K., Hafez M.M. (2021). Eugenol-Induced Autophagy and Apoptosis in Breast Cancer Cells via PI3K/AKT/FOXO3a Pathway Inhibition. Int J Mol Sci. 22 (17), 9243. doi: 10.3390/ijms22179243

Chen R.Z., Yang F., Zhang M., Sun Z.G., Zhang N. (2021). Cellular and Molecular Mechanisms of Pristimerin in Cancer Therapy: Recent Advances. Front Oncol. 11, 671548. https://doi.org/10.3389/fonc.2021.671548

Kim U., Kim C.Y., Lee J.M., Oh H., Kim J., Park J.H. (2020). Phloretin Inhibits the Human Prostate Cancer Cells Through the Generation of Reactive Oxygen Species. Pathol Oncol Res. 26(2), 977-984. doi: 10.1007/s12253-019-00643-y

Li N., Yang F., Liu D.Y., Guo J.T., Ge N., Sun S.Y. (2021). Scoparone inhibits pancreatic cancer through PI3K/Akt signaling pathway. World J Gastrointest Oncol. 13 (9), 1164-1183. doi: 10.4251/wjgo.v13.i9.1164

Liu J., Liu X., Ma W., Kou W., Li C., Zhao J. (2018). Anticancer activity of cucurbitacin-A in ovarian cancer cell line SKOV3 involves cell cycle arrest, apoptosis and inhibition of mTOR/PI3K/Akt signaling pathway. J BUON. 23 (1), 124-128

Mahendra C.K., Tan L.T.H., Lee W.L., Yap W.H., Pusparajah P., Low L.E., et al. (2020). Angelicin-A Furocoumarin Compound with Vast Biological Potential. Front Pharmacol. 11, 366. doi: 10.3389/fphar.2020.00366

Sánchez M., González-Burgos E., Iglesias I., Gómez-Serranillos M.P. (2020). Pharmacological Update Properties of Aloe Vera and its Major Active Constituents. Molecules. 25 (6), 1324. doi: 10.3390/molecules25061324

Sp N., Kang D.Y., Lee J.M., Bae S.W., Jang K.J. (2021). Potential Antitumor Effects of 6-Gingerol in p53-Dependent Mitochondrial Apoptosis and Inhibition of Tumor Sphere Formation in Breast Cancer Cells. Int J Mol Sci. 22 (9), 4660. doi: 10.3390/ijms22094660

Wang J., Ma Y., Guo M., Yang H., Guan X. (2021). Salvianolic acid B suppresses EMT and apoptosis to lessen drug resistance through AKT/mTOR in gastric cancer cells. Cytotechnology. 73 (1), 49-61. doi: 10.1007/s10616-020-00441-4

Wang X., Bai Y., Yan X., Li J., Lin B., Dai L., et al. (2021). Cucurbitacin B exhibits antitumor effects on CD133+ HepG2 liver cancer stem cells by inhibiting JAK2/STAT3 signaling pathway. Anticancer Drugs. 32 (5), 548-557. doi: 10.1097/CAD.0000000000001062

Yang P., Liu W., Fu R., Ding G.B., Amin S., Li Z. (2020). Cucurbitacin E Chemosensitizes Colorectal Cancer Cells via Mitigating TFAP4/Wnt/β-Catenin Signaling. J Agric Food Chem. Epub ahead of print. doi: 10.1021/acs.jafc.0c05551

Zhang J.M., Jiang Y.Y., Huang Q.F., Lu X.X., Wang G.H., Shao C.L. (2021). Brefeldin A delivery nanomicelles in hepatocellular carcinoma therapy: Characterization, cytotoxic evaluation in vitro, and antitumor efficiency in vivo. Pharmacol Res. 172, 105800. doi: 10.1016/j.phrs.2021.105800

Zhang N., Hua X., Tu H., Li J., Zhang Z., Max C. (2021). Isorhapontigenin (ISO) inhibits EMT through FOXO3A/METTL14/VIMENTIN pathway in bladder cancer cells. Cancer Lett. 520, 400-408. doi: 10.1016/j.canlet.2021.07.041

Zhou X., Wang W., Li Z., Chen L., Wen C., Ruan Q., Xu Z., et al. (2022). Rosmarinic Acid Decreases the Malignancy of Pancreatic Cancer Through Inhibiting Gli1 Signaling. Phytomedicine. 95, 153861. doi: 10.1016/j.phymed.2021.153861
